# Molecular mechanism of targeted inhibition of HMGA2 via miRNAlet-7a in proliferation and metastasis of laryngeal squamous cell carcinoma

**DOI:** 10.1042/BSR20193788

**Published:** 2020-06-03

**Authors:** Li-Juan Ma, Jun Wu, En Zhou, Juan Yin, Xu-Ping Xiao

**Affiliations:** Department of Otolaryngology Head/Neck Surgery, Hunan Provincial People’s Hospital, The First Affiliated Hospital of Hunan Normal University, Changsha 410005, P. R. China

**Keywords:** cell apoptosis, cell proliferation, HMGA2, laryngeal squamous cell carcinoma, MiRNAlet-7a

## Abstract

MiRNAlet-7a is associated with the tumorigenesis of laryngeal squamous cell carcinoma (LSCC). Our study was designed to infer whether let-7a targets high-mobility AT-hook 2 (HMGA2) and suppresses laryngeal carcinoma cell proliferation, invasion, and migration. The expression levels of let-7a and HMGA2 were measured in 30 LSCC clinical specimens by qRT-PCR and their correlation was analyzed. Cell model and mice xenograft model with or without let-7a overexpression were constructed to evaluate the effects of let-7a on LSCC. Moreover, luciferase assay was performed to reveal the interaction between let-7a and HMGA2, which was further verified in xenograft. Let-7a was significantly down-regulated and HMGA2 was up-regulated in LSCC tissues compared with normal tissues (*P<*0.05), both of which were significantly correlated with TNM stage and lymph node metastases of LSCC patients (*P<*0.05). We also observed a negative correlation between let-7a and HMGA2 expression in LSCC samples (*r* = −0.642, *P<*0.05). *In vitro* and *in vivo* experiments demonstrated that let-7a overexpression could inhibit cell proliferation and tumor growth of LSCC and simultaneously down-regulate the expression of HMGA2. Moreover, the regulation of HMGA2 by let-7a was also proved by luciferase assay. Our results revealed that let-7a promotes development and progression of LSCC through inhibiting the expression of HMGA2. Therefore, let-7a may thus be a potential diagnostic biomarker and therapeutic target for treating LSCC.

## Introduction

Laryngeal squamous cell carcinoma (LSCC) is a common type of head and neck cancer, which accounts for 5.7–7.6% of all malignant tumors [1]. Although progress has been made in the treatment of LSCC, long-term survival of LSCC patients is still far from satisfactory, which may be resulted from the largely unclear etiology. Multiple carcinogenic factors have been found to be involved in occurrence and development of LSCC. Therefore, deepening the understanding of molecular and genetic pathogenesis of LSCC could promote the development of novel and more effective treatment strategy for LSCC, and is of great significance for LSCC patients

Previously, we had built and studied the whole genome expression profiles of LSCC at different clinical stages and identified various molecular markers. Among them, high-mobility group A2 (HMGA2), whose expression was significantly up-regulated, specifically attracted our attention [2]. The *HMGA2* gene is located on chromosome 12q13–15 and encodes HMGA2 protein containing 109 amino acids. It has been recognized as a new oncogene that may contribute to tumorigenesis, invasiveness, and metastasis. Studies showed that its transcripts were hardly detected in late stages of embryonic development or in completely differentiated mature cells and tissues. However, it was highly expressed in many malignant and benign tumors, such as nasopharyngeal carcinoma [3], pancreatic [4], gastric [5], colon [6], and ovarian cancers [7]. Thus, HMGA2 may be a useful marker for cancer diagnosis and treatment, as well as elucidating the biological behavior and prognosis of tumors.

Recently, a relationship between HMGA2 and microRNA (miRNA) in tumors has been reported [8,9]. MiRNA, a single-stranded RNA composed of ∼22 nucleotides, contributes to regulation of target gene by inhibiting protein translation and regulating endogenous gene expression via incomplete complementary pairing with target mRNA. Let-7 is an important member of the miRNA family. Recent studies suggested that let-7 could regulate the expression of a variety of oncogenes that contribute to carcinogenesis in liver [10], ovarian [11], esophageal [12], oral cancers [13], and head and neck tumors [14]. In a word, let-7 has been recognized as a tumor suppresser. On the other hand, studies indicated that let-7a, which exhibits similar effects on human cancers with let-7, could combine the 3′-UTR of the proto-oncogene c-MYC [15], HMGA2 [16], and RAS [17]. However, the molecular mechanism underlying the regulation of proliferation, invasion and migration of LSCC by let-7a/HMGA2 axis is still largely clear.

In the present study, we showed that let-7a was down-regulated and HMGA2 was up-regulated in LSCC tissues compared with normal tissues, which were both associated with clinical TNM stage and lymph node metastases. Furthermore, there was an inverse correlation between expression of let-7a and HMGA2 in LSCC patients. Let-7a mimics inhibited proliferation and invasion of LSCC cells by targeting HMGA2 *in vitro*. Furthermore, let-7a could suppress tumor growth *in vivo*. Therefore, we offer evidence that let-7a may be a diagnostic marker and a therapeutic target for LSCC.

## Materials and methods

### Clinical specimens

A total of 30 freshly frozen laryngeal carcinoma tissues and paracarcinoma normal tissues were collected from Hunan Provincial People’s Hospital between March 2015 and March 2017. No patients received tumor-specific therapy before diagnosis. Informed consent was obtained from every patient, and this was approved by the Institutional Ethics Committee of Hunan Provincial People’s Hospital. Histopathological diagnosis was diagnosed by two pathologists. Clinical staging was based on the 7^th^ edition of the AJCC Cancer Staging Manual. Data for demographic and clinicopathological characteristics appear in Table 1.

**Table 1:** The clinicopathologic characteristics of let-7a and HMGA2 in LSCC

Characteristics	*n*	Let-7a		*P*	HMGA2		*P*
		High, *n* (%)	Low, *n* (%)		High, *n* (%)	Low, *n* (%)	
**Gender**							
Male	28	8 (28.6)	20 (71.4)	0.523	12 (42.9)	16 (57.1)	0.844
Female	2	1 (50.0)	1 (50.0)		1 (50.0)	1 (50.0)	
**Age (y)**							
≥60	22	9 (40.9)	13 (59.1)	0.866	10 (41.7)	12 (58.3)	0.8
<60	8	3 (37.5)	5 (62.5)		4 (50.0)	4 (50.0)	
**TNM stage**							
I-II	16	12 (75.0)	4 (25.0)	0.030	5 (31.2)	11 (68.8)	0.0
III-IV	14	5 (35.7)	9 (64.3)		10 (71.4)	4 (28.6)	
**Lymph node metastasis**							
No	12	8 (66.6)	4 (33.3)	0.015	5 (41.7)	7 (58.3)	0.0
Yes	18	4 (22.2)	14 (78.8)		15 (83.3)	3 (16.7)	

### Experimental cells and animals

TU212 cells were purchased from Shanghai cell institute, Chinese academy of sciences. Male BALB/C mice (*N*=18, 4–6 weeks of age, 10–20 g) were purchased from Nanjing Biomedical Research Institute of Nanjing University and housed in an SPF environment (constant temperature 18–22°C, constant humidity 50–80%) in the Teaching Center of Biomedical Experiments, Hunan Normal University. Mice were randomized to three groups (*N* = 6/group): blank (TU212), NC (TU212 /NC), and let-7 groups (TU212 /let-7a).

### Cell culture

TU212 cells were cultured in 1640 medium containing 10% fetal bovine serum (Shanghai ExCell Biology, Inc.) and 1% penicillin–streptomycin and placed in an incubator at 37°C, 5% CO_2_, saturated humidity. When the cell density reached ∼80%, cells were digested with trypsin and 1640 medium was added (containing 10% FBS) to the subculture.

### Cell transfection

Transfection was performed with the cationic liposome method according to instructions for Lipofectamine 2000 reagent (Life Technologies, Shanghai). After 6 h, medium containing lip2000 was removed and fresh medium was replaced. Transfection was observed, photographed, and calculated under an inverted fluorescent microscope. Transfection efficiency of FAM-labeled human let-7a mimics (GenePharma, Shanghai) was observed in the same manner.

### Apoptotic assay

High expression of let-7a and TU212 cell apoptosis was analyzed with an Annexin V-FITC apoptosis detection kit. Cells were observed under fluorescent microscopy or flow cytometry for 1 h.

### MTT assay

Cell proliferation was analyzed using an MTT assay. Briefly, 1 × 10^3^ cells were seeded into a 96-well plate in quadruplicate for each condition. Cells were incubated for 12, 24, 48 and 72 h. About 20 μl of MTT (5 mg/ml) (Sigma, location) was added to each well and incubated for 4 h. At the end of incubation, supernatants were removed and 150 μl of DMSO (MP, location) was added to each well. OD value was read for each well at 490 nm.

### Luciferase assay

The wild-type and mutant HMGA2 3′-untranslational region (UTR) luciferase reporters were obtained from Shanghai GenePhama Co., Ltd. (Shanghai, China). Human HMGA2 cDNA 3′-UTR region and mutated 3′-UTR region were generated with genomic DNA from 239T cells using PCR with the following primers as shown in the Supplementary Material, and then cloned into the XhoI and NotI sites of pmiR-RB-REPORT™ vector (Promega, Madison, WI, U.S.A.). After amplification and DNA-sequence confirmation, these vectors were named as pmiR-HMGA2-3′-UTR-wt and pmiR-HMGA2-3′-UTR-mut, respectively. For luciferase assay, HEK-293 cells were cultured and co-transfected with these luciferase reporters and let-7a mimic or a negative control vector for 36 h using Lipofectamine 2000 (Invitrogen). Subsequently, cells were subjected to protein extraction and the dual luciferase assay system (Promega) to measure the luciferase activity according to the kit instructions. The luciferase activity of each sample was then normalized to Renilla luciferase activity.

### EdU Staining

Proliferation of cells was cytochemically detected according to the manufacturer’s instructions (C10310-3, Ribobio, Guangzhou, China). In brief, the tumor cells were incubated with the EdU staining buffer, fixed in 4% polyformaldehyde, and stained the nuclear with DAPI. The stained cells were observed and photographed under microscope. In addition, integrated optical density (IOD) was used to assess EdU-positive cells with ImageJ software (Bethesda, MD, U.S.A.).

### qRT-PCR

qRT-PCR was performed according to kit instructions (Invitrogen, Carlsbad, CA). Target genes were reverse transcribed with ploy(A) tailing; obtained reverse transcripts were added to primers for let-7a and U6 and HMGA2 and β-actin (provided by Whitehead/Massachusetts Institute of Technology Center for Genome Research, Cambridge, MA) and a fluorescent dye. The reaction was pre-denaturation at 95°C, 10 min, denaturation at 95°C, 10 s, annealing and extension at 59°C, 50 s for 40 cycles total. After the reaction was completed, software automatically performed data analysis, adjusted the baseline, and calculated *C*_t_ values. Quantification was done using the 2^−ΔΔCt^ method. The U6 primer sequence was 5′-GCTTCGGCAGCACATATACTAAAAT-3′; the let-7a primer sequence was 5′-CTATACAACCTACTGCCTTCCC-3′; the HMGA2-F primer sequence was 5′-CAGCCCAGGGACAACCT-3′; the HMGA2-R primer sequence was 5′ -CTGCCTCTTGGCCGTTT-3′ and the amplified fragment length was 196 bp. The primer sequence for β-actin-F was 5′- CATCCTGCGTCTGGACCTGG-3′; the primer sequence of β-actin-R was 5′-TAATGTCACGCACGATTTCC-3′ and the amplified fragment length was 107 bp.

### H&E staining and IHC

Tumors were halved and one half was immersed in formalin solution overnight. After normal paraffin embedding, serial histological sectioning and H&E staining was carried out. The other half of tissue was placed in a cryo-tube and stored in liquid nitrogen for subsequent mRNA and protein extraction. Immunohistochemical (IHC) assay was performed by SP method, and the operation was carried out strictly according to the kit instructions.

### Western blot

Protein was measured according to the instructions in the BCA Protein Quantification Kit (Wellbio, China). Briefly, 50 μg of protein sample was resolved with SDS-PAGE and transferred to a PVDF membrane using electroblotting. Blots were blocked with 5% skimmed milk for 1 h; incubated with primary antibody HMGA2 (1:1000; Proteintech) in a shaker at 4°C overnight. Membranes were washed with TBST and incubated with secondary antibody at room temperature for 1 h. Membranes were washed again with TBST and ECL was used to visualize bands. HMGA2 protein expression was normalized to β-actin (1:4000 Proteintech) as an internal reference. OD was calculated for each gray band using Quantity One software. Relative expression of HMGA2 = gray HMGA2 band/gray β-actin band.

### Animal model

All animal experiments were taken place in Animal Experimental Center of Medical University School of Hunan Normal University. TU212 cells were transfected with let-7a mimics and let-7a NC and residual medium was removed (10% FBS) with PBS washing. Then, cells were digested in 0.25% trypsin and cell volume was adjusted with RPMI1640 medium (serum free) to obtain a cell density of 2.5 × 10^7^ cells/ml. Cells were injected (0.2 ml suspension; 5 × 10^6^ cells, sc) in the right back sides of nude mice. Mice in let-7a group were given let-7a mimic cells and let-7a NC cells were used for the NC group. TU212 cells were used for blank controls. Intraperitoneal injection of sodium pentobarbital was performed for anesthesia (60 mg/kg) and euthanasia (120 mg/kg), respectively.

### Mice xenograft

Mice were observed for feeding and activity from the day of inoculation. Tumors were measured and recoded as was animal health. After tumors formed, major (*b*) and minor (*a*) diameters were measured every 3 days. Tumor volume was calculated as follows: *V* = *ab*^2^/2. Growth curves were drawn after the 30-day observation. Animals were killed on the 30^th^ day post-inoculation and tumors were dissected, weighed and assessed for quality and size. Tumor growth inhibition was calculated as follows: (%) = (1 − average tumor weight of experimental group/average tumor weight of control group) × 100%.

### Statistical methods

Data were assessed with SPSS23.0. Measurement data normally distributed with equal variances are reported as means + SD and a Student’s *t* test or ANOVA was used to assess statistical significance. For data with unequal variances, comparisons between groups was performed with a Mann–Whitney *U* test; comparisons among multiple groups was made with a Kruskal–Wallis *H* test (*α* = 0.05). *P<*0.05 represented significant differences.

## Results

### The relationship between Let-7a and HMGA2 expression with clinicopathological characteristics of LSCC patients

**Figure 1:**
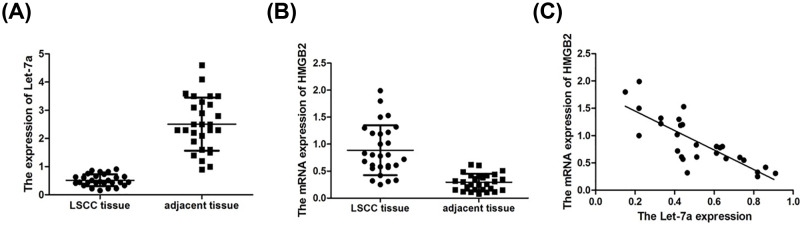
Let-7a and HMGA2 expression and the relationship with clinicopathological characteristics in LSCC patients (**A**) Expression of let-7a and HMGA2 in LSCC clinical samples. (**B**) Let-7a was down-regulated and HMGA2 was up-regulated in LSCC (*n*=30) compared with paracarcinoma normal tissues. (**C**) Significant correlations between expression of let-7a and HMGA2 in LSCC were demonstrated with Pearson’s correlation coefficient analysis (*r* = −0.642, *P*=0.008).

For preliminarily exploring the role of let-7a and HMGA2 in LSCC, the expression levels were detected 30 freshly frozen LSCC tissues compared with paracarcinoma normal tissues. The results showed that let-7a expression was lower and HMGA2 expression was greater in tissue samples (Figure 1A,B; *P<*0.05). The statistical analysis of correlation between let-7a and HMGA2 and LSCC patient characteristics were showed in Table 1. In LSCC patients, the expression of let-7a was negatively correlated with HMGA2 in LSCC (*r* = −0.642, *P*=0.008; Figure 1C).

### Let-7a inhibits LSCC development *in vitro*

**Figure 2:**
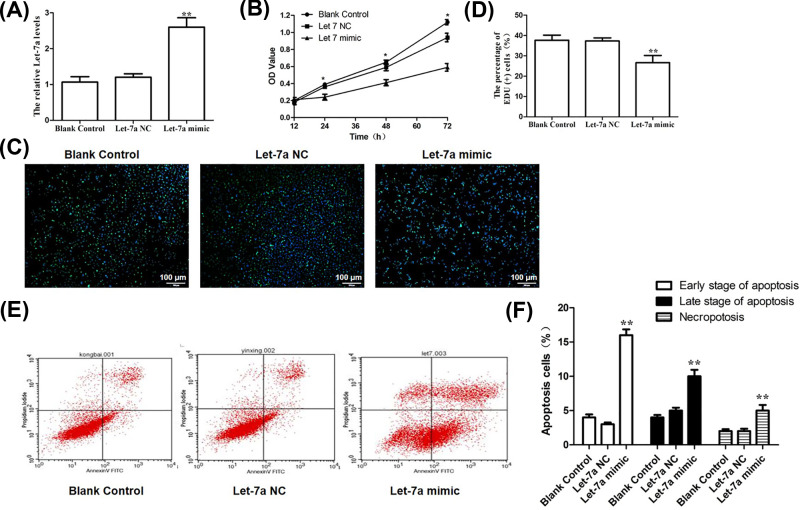
Let-7a inhibits laryngeal carcinoma growth *in vitro* (**A**) Relative expression of let-7a miRNA in laryngeal carcinoma cells. (**B**) Growth curves determined by MTT assay showed that let-7a mimics inhibited cell proliferation compared with NC and blank groups (*P*<0.05). (**C**) The merge of EdU staining (Green) and Hoechst 33342 staining (Blue) for laryngeal carcinoma TU212 cells after transfection of let-7a mimics. (**D**) The semi-quantitative analysis of the percentage of EdU positive cells; Mean ± SD, *n*=3, *P<0.05, **P<0.01 versus Blank control group or let-7a NC group. (**E** and **F**) Apoptosis of TU212 cells measured by flow cytometry. After transfection of let-7a mimics, apoptosis was significantly enhanced and this was statistically significant compared with the other two groups (*P<*0.05).

In order to illustrate the regulatory role of let-7a in LSCC, TU212 cells with overexpression of let-7a were constructed and confirmed by qRT-PCR (Figure 2A)*.* Growth curves determined by MTT assay showed that let-7a overexpression inhibited cell growth compared with blank and NC groups at 72 h (*P<*0.05; Figure 2B). It was also confirmed by the merge of EdU staining (Green) and Hoechst 33342 staining (Blue) for TU212 cells after transfection of let-7a mimics. As shown in Figure 2C,D, the proportion of EdU-positive cells in TU212 cells with overexpressed let-7a were decreased significantly compared with blank and NC groups. Moreover, we also observed promoted apoptosis of TU212 cells 24 h after transfection of let-7a mimics (Figure 2E,F).

### HMGA2 was identified as the let-7a-targeting gene in LSCC cancer cells

**Figure 3:**
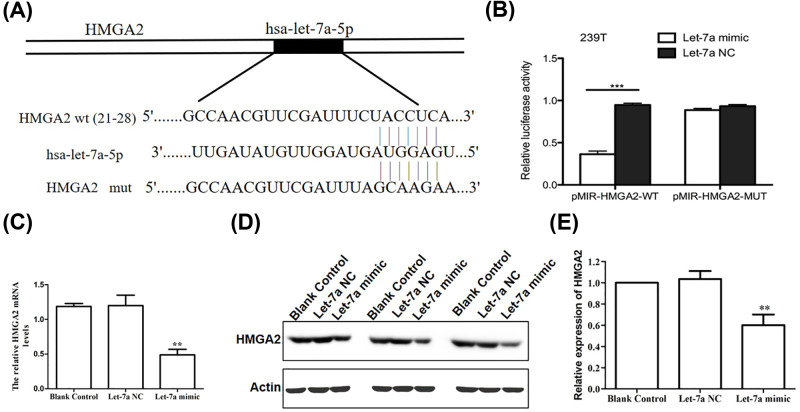
HMGA2 as the target of let-7a in LSCC cells (**A**) Illustration of let-7a targeting sequences of HMGA2 3′-UTR or mutations containing six mutant sites in HMGA2 3′-UTR used for luciferase assay. (**B**) Luciferase activity. HEK239T cells were transfected with let-7a site mutant 3′-UTR-driven reporter constructs; wt, wild-type (*n*=3). (**C**) Relative expression of HMGA2 mRNA in the laryngeal carcinoma cells was detected by RT-qPCR. (**D**) The images of Western blot for determining expression of HMGA2 protein in the laryngeal carcinoma cells. (**E**) The relative ratio of HMGA2/actin in laryngeal carcinoma cells; Mean ± SD, *n*=3, **P<0.01, ***P<0.001 versus Blank control group or let-7a NC group.

Subsequently, we explored the molecular mechanism by which let-7a suppressed LSCC development. Gene target analysis was performed using online tools (miRanda and miRbase) to identify potential let-7a-binding genes and found that let-7a binds to *HMGA2* 3′-UTR region (Figure 3A). We then constructed a luciferase reporter plasmid carrying *HMGA2* 3′-UTR (Figure 3B). The luciferase activity was significantly decreased in the Luc-*HMGA2*-3′-UTR-transfected cells compared with that of the potential target site of mutant let-7a in *HMGA2* 3′-UTR and negative control cells. We analyzed HMGA2 mRNA expression using qRT-PCR inTU212 cells. HMGA2 mRNA in blank and NC groups was significantly greater than the let-7a mimics group (*P*<0.05) (Figure 3C). HMGA2 protein expression measured with Western blot (Figure 3D,E) showed that HMGA2 was down-regulated in let-7a group compared with blank and NC groups (*P*<0.05).

### Let-7a inhibits LSCC development *in vivo*

**Figure 4:**
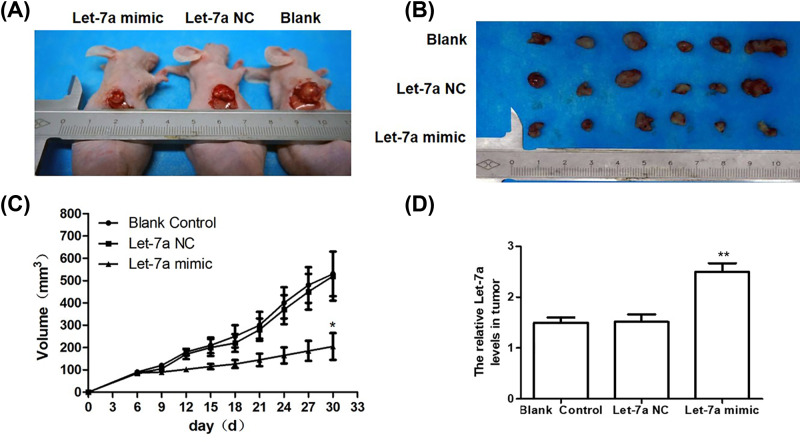
Let-7a inhibits laryngeal carcinoma growth *in vivo* (**A**) Size of *in vivo* xenografts. (**B**) Size of *ex vivo* xenografts. Xenograft in the let-7a group was significantly smaller than in the other two groups (*P<*0.01). (**C**) The growth curve of the xenografts. Transfection of let-7a mimics significantly inhibited tumor growth (*P<*0.01). (**D**) The expression of let-7a miRNA in the xenografts; Mean ± SD, *n*=6. *P<0.05, **P<0.01 versus Blank control group or let-7a NC group.

Then, mice xenograft model was constructed through the injection of LSCC cells with or without let-7a overexpression. The xenograft tumors were extracted and representative tumor morphologies were shown in Figure 4A,B. Isolated tumors from animal models were multinodular with a ‘fish flesh’ appearance and no ulcerations on surfaces. Pairwise comparisons discovered no significant difference between blank and NC groups, indicating the ignorable effects of let-7a NC (*t* = 0.498, *P*=0.360). In consistent with previous *in vitro* studies, the volume of tumors removed from let-7a mimic group was significantly smaller than the other two groups (Figure 4C, *P*<0.05). Furthermore, the elevation of let-7a expression in let-7a mimic group was confirmed by qRT-PCR (Figure 4D, *P*<0.01), confirming the inhibition of LSCC by let-7a.

### HMGA2 mRNA and protein expression *in vivo*

**Figure 5:**
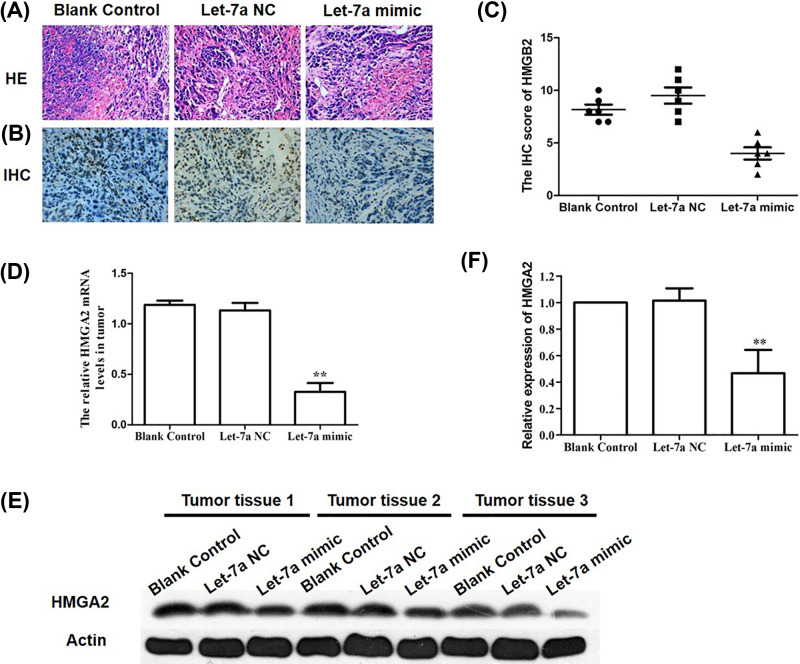
HMGA2 mRNA and protein expression *in vivo* (**A**) H&E staining of nude mouse xenografts after inoculation of TU212 cells (400×). Tumors had enlarged nuclei; nucleolus had distinct atypical cells, suggesting successful establishment of mice xenograft model. (**B** and **C**) IHC analysis for the protein expression of HMGA2 in the xenografts. (**D**) Expression of HMGA2 mRNA in the xenografts. (**E**) The images of Western blot for determining the protein expression of HMGA2 in the xenografts. (**F**) The relative ratio of HMGA2/actin in the xenografts; Mean ± SD; *n*=6. **P<0.01 *vs* Blank control group or let-7a NC group.

H&E staining of tumors on the right neck and back of nude mice showed enlarged nuclei with distinct atypical cells, and this was consistent with characteristics of TU212 cell xenografts and confirmed xenograft establishment (Figure 5A). The HMGA2 protein expression was examined and showed that the signal of HMGA2 mainly located in the nucleus, and exhibited high expression as detected by IHC (deep to brown) (Figure 5B,C). On the other hand, the results of qRT-PCR demonstrated that HMGA2 mRNA level in blank and NC groups was significantly greater than the let-7a mimics group (*P<*0.05, Figure 5D). The down-regulated protein level of HMGA2 was also observed in tumors removed from let-7a group compared with the other groups (Figure 5E,F). Since HMGA2 was significantly decreased *in vivo* and *in vitro*, it could be deduced that HMGA2 may be a target of let-7a.

## Discussion

Let-7 is reported to be capable of inhibiting the formation of microvessels around tumors, resulting in a hypoxic microenvironment and inhibition of tumor growth and metastasis [18]. HMGA2 is a non-histone chromosomal protein that guarantees the independence and migration of cancer cells. Studies have indicated that HMGA2 is an oncogene that promotes chromosomal translocation and up-regulates transcription. Its expression is up-regulated in various types of tumors and is positively associated with tumor severity and invasiveness [19,20]. Recent studies suggested that several let-7 family members have diminished expression in cancer compared with normal tissues, and the effects of them on migration and invasiveness of cancer cells could be blocked by HMGA2, Lin28, or MMPs [21–23]. Moreover, HMGA2 was up-regulated in glioma tissues than in normal brain tissues, suggesting its potential role as a functional target of let-7a via binding of the 3′-UTR [24]. In a word, let-7a may have anti-tumor effects via targeting HMGA2 but whether this may work in LSCC is unclear.

We found that expression of let-7a was down-regulated in LSCC tissues compared with normal tissues, and let-7a expression was negatively correlated with clinical TNM stage and lymph node metastases of LSCC patients. HMGA2 was up-regulated in LSCC samples and was positively related to TNM stage and lymph node metastases. There was a negative correlation between let-7a expression and HMGA2 expression in LCSS patients and this was in consistent with the previously reported results in oral squamous cell carcinoma [13], neuroendocrine tumors [25], and lung cancer [17]. Thus, let-7a may regulate LSCC cell invasion and metastasis by targeting HMGA2.

In our study, let-7a mimics were transfected into TU212 cells to induce high expression of let-7a, and then cell proliferation and apoptosis were measured. The results clarified that overexpression of let-7a significantly inhibited cell proliferation and promoted cell apoptosis *in vitro*. The inhibition of LSCC by let-7a overexpression was also confirmed by *in vivo* studies. These data agreed with the published work by Li et al. [24], which demonstrated that let-7a inhibited tumor growth in nude mice.

On the other hand, the expression of let-7a and HMGA2 detected by qPCR showed a negative correlation between them, suggesting the targeted inhibition of HMGA2 by let-7a. Similar results could also be seen in esophageal [26], thyroid [27], and colorectal cancer [28]. Likely, when acidic carboxylic tails including 3′-UTRs are lost during gene rearrangement of HMGA2, its targeted inhibition by let-7a is lost. Moreover, it was previously reported that abnormal activation of HMGA2 could promote tumorigenesis [29–31]. Using a series of HMGA2 expression structures to study lung cancer, Kumar’s group verified that the 3′-UTR of HMGA2 can be used as a ceRNA of the TGF-β co-receptor tgfbr3. Through competitive binding, it can inhibit miRNA let-7, enhance the TGF-β signaling pathway, lead to EMT and pluripotency, and ultimately promote proliferation and metastasis of lung cancer cells [32]. Therefore, more complex mechanisms may exist between let-7a and HMGA2 in LSCC.

In summary, let-7a regulated LSCC cell proliferation, invasion, and metastasis through targeting HMGA2, which may be a therapeutic approach for treating LSCC.

## Supplementary Material

Supplementary Material

## Abbreviations

HMGA2: high-mobility AT-hook 2 EMT
LSCC: laryngeal squamous cell carcinoma
miRNA: microRNA

## References

[1] Wang Q., Liu Y., Hu G. et al. (2016) The survival rate and larynx preservation in elderly cancer patients who received surgical operation: A retrospective cohort study. Int. J. Surg. 36, 342–346 10.1016/j.ijsu.2016.11.073 27871805

[2] Ma Li-Juan, Li Wei, Zhang Xin et al. (2009) Differential Gene Expression Profiling of Laryngeal Squamous Cell Carcinoma by Laser Capture Microdissection and Complementary DNA Microarrays. Arch. Med. Res. 40, 114–123 10.1016/j.arcmed.2008.12.005 19237021

[3] Xia Y.Y., Yin L., Tian H. et al. (2015) HMGA2 is associated with epithelial-mesenchymal transition and can predict poor prognosis in nasopharyngeal carcinoma. Onco. Targets Ther. 8, 169–176 10.2147/OTT.S74397 25653540 PMC4303461

[4] Piscuoglio S., Zlobec I., Pallante P. et al. (2012) HMGAl and HMGA2 protein expression correlates with advanced tumour grade and Iymph node metastasis in pancreatic adenocarcinoma. Histopathology 60, 397–404 10.1111/j.1365-2559.2011.04121.x 22276603

[5] Zha L., Zhang J., Tang W. et al. (2013) HMGA2 elicits EMT by activating the Wnt-catenin pachway in gastric cancer. Dig. Dis. Sci. 58, 724–733 10.1007/s10620-012-2399-6 23135750

[6] Li Y., Zhao Z., Xu C. et al. (2014) HMGA2 induces transcription factor Slug expression to promote epithelial-to-mesenchymal transition and contributes to colon cancer progression. Cancer Lett. 355, 130–140 10.1016/j.canlet.2014.09.007 25218351

[7] Hetland T.E., Holth A., Kam J. et al. (2012) HMGA2 protein expression in ovarian serous carcinoma effusions, primary tumors, and solid metastases. Virchows. Arch. 460, 505–513, Format 10.1007/s00428-012-1228-9 22476403

[8] Yan J., Zhang Y., Shi W. et al. (2016) The critical role of HMGA2 in regulation of EMT in epithelial ovarian carcinomas. Tumor Biol 37, 1–6 10.1007/s13277-015-3852-x 26250458

[9] Zhao X.P., Zhang H., Jiao J.Y. et al. (2016) Overexpression of HMGA2promotes tongue cancer metastasis through EMT pathway. J. Transl. Med. 14, 1–11 10.1186/s12967-016-0777-0 26818837 PMC4730598

[10] Tang H., Zhang P., Xiang Q. et al. (2014) Let-7 g microRNA sensitizes fluorouracil resis-tant human hepatoma cells. Pharmazie 69, 287–292 24791593

[11] Wendler A., Keller D., Albrecht C. et al. (2011) Involvement of let-7/miR-98 microRN-As in the regulation of progesterone receptor men'lbrane component 1 expression in ovarian cancer cells. Oncol. Rep. 25, 273–279 21109987

[12] Liu Q., Lv G.D., Qin X., Gen Y.H., Zheng S.T., Liu T. et al. (2012) Role of microRNA let-7 and effect to HMGA2 in esophageal squamous cell carcinoma. Mol. Biol. Rep. 39, 1239–1246 10.1007/s11033-011-0854-7 21598109

[13] Sterenczak K.A., Eckardt A., Kampmann A. et al. (2014) HMGA1 and HMGA2 expressi-on and comparative analyses of HMGA2, Lin28 and let-7 miRNAs in oral squa-mous cell carcinoma. BMC Cancer 23, 694–705 10.1186/1471-2407-14-694 PMC4190370 25245141

[14] Yamazaki H., Mori T., Yazawa M. et al. (2013) Stem cell self-renewal factors Bmi1 and HMGA2 in head and neck squamous cell carcinoma: clues for diagnosis. Lab. Invest. 93, 1331–1338 10.1038/labinvest.2013.120 24145240

[15] Li Y., Liu H., Lai C. et al. (2013) The Lin28/let-7a/c-Myc pathway plays a role in non-muscle invasive bladder cancer. Cell Tissue Res. 354, 533–541 10.1007/s00441-013-1715-6 24036903

[16] Xu C., Sun X., Qin S. et al. (2015) Let-7a regulates mammosphere formation capacity through Ras/NF-κB and Ras/MAPK/ERK pathway in breast cancer stem cells. Cell Cycle 14, 1686–1697 10.1080/15384101.2015.1030547 25955298 PMC4615052

[17] Wang Y.Y., Ren T., Cai Y.Y. et al. (2013) MicroRNA let-7a inhibits the proliferation and invasion of nonsmall cell lung cancer cell line 95D by regulating K-Ras and HMGA2 gene expression. Cancer Biother. Radiopharm. 28, 131–137 10.1089/cbr.2012.1307 23134218

[18] da Silva A.P., Feliciano T., Vaz Freitas S. et al. (2014) Quality of Life in Patients Submitted to Total Laryngectomy. J. Voice 9, 1–7 10.1016/j.jvoice.2014.09.002 25619472

[19] Ma C., Nong K., Zhu H. et al. (2014) H19 promotes pancreatic cancer metastasis by\ derepressing let-7’S suppression on its target HMGA2-mediated EMT. Tumour Biol. 35, 9163–9169 10.1007/s13277-014-2185-5 24920070

[20] Natarajan S., Hombach-Klonisch S., Droge P. et al. (2013) HMGA2 inhibits apoptosis through interaction wilh ATR·CHKl signaling complex in human cancer cells. Neoplasia 15, 263–280 10.1593/neo.121988 23479505 PMC3593150

[21] Lee H., Han S., Kwon C.S. et al. (2016) Biogenesis and regulation of the let-7 miRNAs and their functional implication. Protein Cell 7, 100–113 10.1007/s13238-015-0212-y 26399619 PMC4742387

[22] Paz E.A., LaFleur B. and Gerner E.W. (2014) Polyamines are oncometabolites that regulate the LIN28/let-7 pathway in colorectal cancer cells. Mol. Carcinog. 53, E96–E106 10.1002/mc.22051 23737330

[23] Qian P., Zuo Z., Wu Z. et al. (2011) Pivotal role of reduced let-7 g expression in breast cancer invasion and metastasis. Cancer Res. 71, 6463–6474 10.1158/0008-5472.CAN-11-1322 21868760

[24] Li Y., Zhang X., Chen D. et al. (2016) Let-7a suppresses glioma cell proliferation and invasion through TGF-β/Smad3 signaling pathway by targeting HMGA2. Tumor Biol. 37, 8107–8119 10.1007/s13277-015-4674-6 26715270

[25] Døssing K.B., Binderup T., Kaczkowski B. et al. (2014) Down-Regulation of miR-129-5p and the let-7 Family in Neuroendocrine Tumors and Metastases Leads to Up-Re-gulation of Their Targets Egr1, G3bp1, Hmga2 and Bach1. Genes (Basel) 6, 1–21 10.3390/genes6010001 25546138 PMC4377830

[26] Liu Q., Liu T., Zheng S. et al., HMGA2 is down-regulated by microRN-A let-7 and associated with epithelial–mesenchymal transition in oesophageal squamous cell carcinomas of Kazakhs. Histopathology 65, 408–417 24612219 10.1111/his.12401

[27] Damanakis A.I., Eckhardt S. et al. (2016) MicroRNAs let7 expression in thyroid cancer: correlation with their deputed targets HMGA2 and SLC5A5. J. Cancer Res. Clin. Oncol. 142, 1213–1220 26960757 10.1007/s00432-016-2138-z PMC11819097

[28] Cheng Y.W. and Chen I. (2014) Abstract 5246: The role of mir-21 and Let-7a in HMGA2 associated colorectal cancer pathogenesis with different APC/k-ras status. Cancer Res. 74, 5246–5246

[29] Guo L., Chen C., Shi M. et al. (2013) Stat3 -coordinated Lin - 28 -let -7 -HMGA2 and mi༲-200 -ZEB1 circuits initiate and main-tain oncostatin M -driven epithelial - mesenchymal transition. Oncogene 32, 5272–5282 10.1038/onc.2012.573 23318420

[30] Erkul E., Yilmaz I., Gungor A. et al. (2016) MicroRNA‐21 in laryngeal squamous cell carcinoma: Diagnostic and prognostic features. Laryngoscope 08, 507–510 10.1002/lary.26226 27545281

[31] Yu X. and Li Z. (2015) The role of MicroRNAs expression in laryngeal cancer. Oncotarget 6, 23297–23305 10.18632/oncotarget.4195 26079642 PMC4695119

[32] Kumar M.S., Armenteros-Monterroso E., East P. et al. (2014) HMGA2 functions as a co-mpeting endogenous RNA to promote lung cancer progression. Nature 505, 212–217 10.1038/nature12785 24305048 PMC3886898

