# Supplementary figures and images for: Molecular mechanism of targeted inhibition of HMGA2 via miRNAlet-7a in proliferation and metastasis of laryngeal squamous cell carcinoma
Source: Biosci Rep. 2020 Jun 3;40(6):BSR20193788. doi: 10.1042/BSR20193788 (PMC7269914; doi:10.1042/BSR20193788)

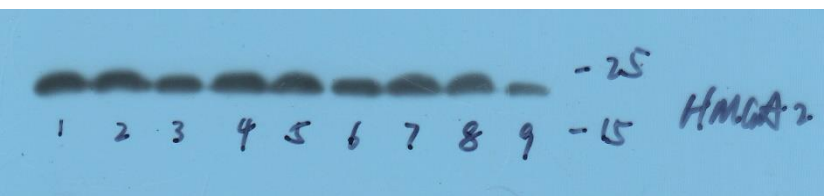

HMGA2-1

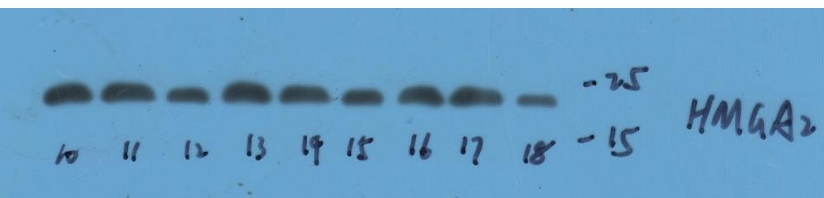

HMGA2-2

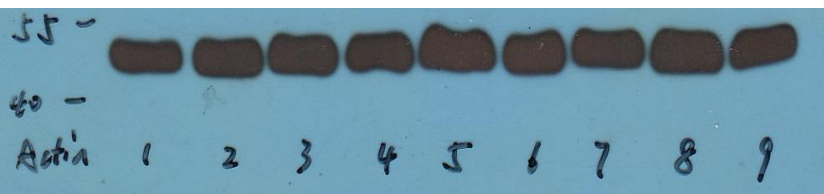

$\beta$ -actin-1

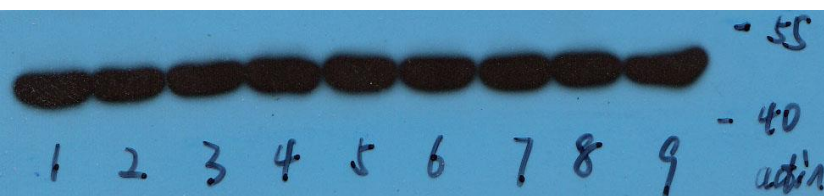

$\beta$ -actin-2

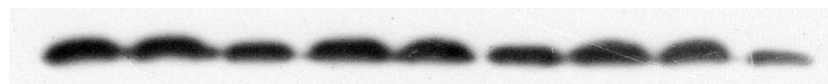

HMGA2-1

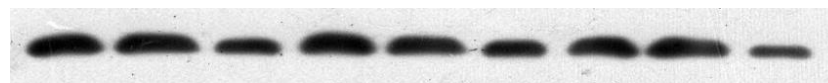

HMGA2-1

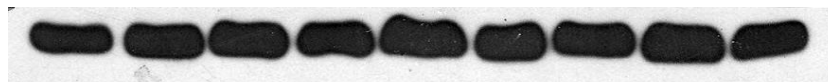

$\beta$ -actin-1

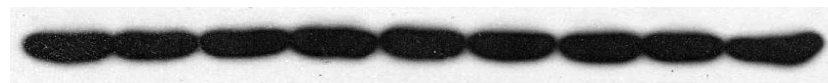

$\beta$ -actin-2

Supplement: Supplementary Material [file BSR-2019-3788_supp.pdf]
